# Mechanisms and outcomes of a very low intensity intervention to improve parental acknowledgement and understanding of childhood overweight/obesity, embedded in the National Child Measurement Programme: A sub‐study within a large cluster Randomized Controlled Trial (MapMe2)

**DOI:** 10.1111/bjhp.12784

**Published:** 2025-02-13

**Authors:** Elizabeth H. Evans, Christopher M. Jones, Ashley Adamson, Angela R. Jones, Laura Basterfield, João Paulo de Aguiar Greca, Letitia Sermin‐Reed, Maddey Patterson, Lorraine McSweeney, Raenhha Dhami, Louisa Ells, Alison Gahagan, Tomos Robinson, Mohadeseh Shojaei Shahrokhabadi, Dawn Teare, Martin J. Tovée, Vera Araújo Soares

**Affiliations:** ^1^ Department of Psychology Durham University Durham UK; ^2^ Department for Prevention of Cardiovascular and Metabolic Diseases, Center for Preventive Medicine and Digital Health (CPD) Medical Faculty Mannheim, Heidelberg University Mannheim Germany; ^3^ Human Nutrition and Exercise Research Centre Population Health Sciences Institute, Newcastle University Newcastle upon Tyne UK; ^4^ School of Health, Obesity Institute Leeds Beckett University Leeds UK; ^5^ Department of Health and Social Care Office for Health Improvement and Disparities London UK; ^6^ Northumbria University Newcastle upon Tyne UK

**Keywords:** child obesity, parental perceptions, weight monitoring

## Abstract

**Objectives:**

Parental underdetection of child underweight and overweight/obesity may negatively affect children's longer‐term health. We examined psychological/behavioural mechanisms of a very low‐intensity intervention to improve acknowledgement and understanding of child weight after feedback from a school‐based weight monitoring programme.

**Design:**

This sub‐study was nested within a larger 3‐arm cluster‐RCT (1:1:1; *N* = 57,300). Parents in all groups received written postal feedback on their child's weight classification. Intervention participants received an enhanced feedback letter with computer‐generated photorealistic images depicting children of different weight classifications, and access to a website about supporting healthy weight, once (intervention one) or twice (intervention two; repeated 6 months after first ‘dose’).

**Methods:**

A quantitative process and outcome evaluation using baseline and 12‐month BMI *z*‐scores of an opt‐in sub‐sample of 502 children aged 4–5 and 10–11. Children completed dietary reports, used accelerometers (MVPA), and self‐reported self‐esteem; 10–11‐year‐olds also self‐reported quality of life and dietary restraint. Parents reported perceptions of child's weight classification, and their intentions, self‐efficacy, action planning and coping planning for child physical activity, dietary intake; parents of 4–5‐year‐olds reported their child's quality of life.

**Results:**

Neither intervention differentially improved parental acknowledgement or understanding of weight classification at follow‐up, although parents in all groups reported better acknowledgement after receiving feedback. The interventions did not affect behavioural/psychological determinants, weight outcomes, children's self‐esteem, dietary restraint or quality of life.

**Conclusions:**

The interventions neither improved parental acknowledgement of child weight, child BMI *z*‐scores and their psychological/behavioural determinants, nor worsened psycho‐social sequelae.

**Statement of contribution**

**What is already known on this subject?**

- Parents do not reliably recognize overweight/obesity in their children.
- Existing interventions to address this to improve child weight outcomes have had mixed results.

**What does this study add?**

- We examined mechanisms and outcomes of an intervention to improve parental weight acknowledgement.
- The intervention did not alter child weight outcomes through the hypothesised mechanisms but nor did it do harm.

## INTRODUCTION

### Parental acknowledgement of child weight

Overweight and obesity in children are prevalent and associated with a range of negative psychosocial and physical sequelae (NCD‐RisC, 2017; Sahoo et al., 2015; Smith et al., 2020). Underweight, whilst less prevalent in higher‐income countries, also carries substantial risks (Abarca‐Gómez et al., 2017; Garrido‐Miguel et al., 2021; NCD‐RisC, 2020). Effective interventions exist to support families in dietary and physical activity behaviour change to help children reach a recommended weight (Ells et al., 2018; Keats et al., 2021; Wehling et al., 2020). However, parents typically perceive their child's weight to be in the recommended range (Lundahl et al., 2014). Consequently, they underestimate the weight status of children with overweight and overestimate the weight status of children with underweight (e.g., Blanchet et al., 2019; Júlíusson et al., 2011). Weight monitoring programmes for children have been established in several countries to not only provide population‐level child weight data but to identify children and families in potential need of support (Davidson et al., 2018). Most (but not all) programmes inform parents of the results of this monitoring, predicated on the premise that awareness of a child's weight status is necessary (albeit not sufficient) for parents to seek help and support with child weight and associated behaviours (e.g., Gerards et al., 2012). The majority of parents informed that their child has a weight higher than the recommended range do not go on to seek professional help (Falconer et al., 2014); rates of help‐seeking for parents of underweight children are unknown.

### Interventions to improve parental acknowledgement of child weight and weight outcomes: Putative mechanisms and concerns about adverse outcomes

To complement weight screening and surveillance programmes, interventions have been developed to increase parental acknowledgement and understanding of child weight, with the aim of ultimately improving weight outcomes. These have had mixed results. Educational interventions have been shown to improve parents' acknowledgement of their child's weight in some (Pakpour et al., 2011; Perrin et al., 2010) but not all (Brown et al., 2020; Rune et al., 2015; Shen et al., 2020) studies. We previously found that adapted weight feedback which included computer‐generated illustrations of the range of child body sizes (presented on paper or via a website) did not change parental acknowledgment but did result in more favourable weight outcomes (at 12 months) in children with overweight whose parents received the adapted feedback compared to controls (Jones et al., 2023: The MapMe1 trial). Sallis et al. (2019) found that a similar feedback letter led to higher uptake of weight management services. However, our previous trial (Jones et al., 2023) is the only study to suggest that interventions of this type can affect weight outcomes. Overall, extant research has involved disparate intervention methods, many not easily scalable (e.g., individual educational classes). There is a need for comprehensive research testing scalable interventions informed by health behavioural theory.

To our knowledge, no previous studies of interventions to improve parental acknowledgement and understanding of child weight in conjunction with weight monitoring feedback have reported in detail on intervention mechanisms or child behavioural/psychological outcomes. Such work is important because it has the potential to reveal intervention mechanisms, indicating how and why the intervention does, or does not, function as intended via psychological and behavioural processes (Michie & Abraham, 2004). It is also important to detect any potential adverse intervention effects (Bonell et al., 2015), and this is particularly the case for interventions involving feedback about child weight. It has previously been argued that both weight monitoring and feedback may harm specific sub‐groups of children's self‐esteem, eating behaviours and quality of life, for example, when a child has a higher body mass index (BMI; Jessen et al., 2023). However, research is mixed and relatively few studies have sought to examine outcomes from the child's perspective. Where parents receive professional advice and support to modify behaviour, negative psychological outcomes appear less, rather than more, likely (Jebeile et al., 2019). An objective empirical study is needed to inform sensitive and evidence‐based public health approaches to parent‐facing communications about weight monitoring results going forward.

### The current study

In this paper we report the results of a study embedded within a larger 3‐arm cluster‐Randomized Controlled Trial (RCT; ISRCTN12378125) of an intervention designed to improve parental acknowledgement and understanding of child weight when receiving postal feedback on their child's weight as part of a national monitoring programme (The National Child Measurement Programme; NCMP). The MapMe2 trial built upon and extended our previous work in MapMe1 (Jones et al., 2023) in a much larger sample of 57,300 children in England. All parents/carers involved in MapMe2 received NCMP feedback on their child's weight, but intervention participants received an enhanced feedback letter including a computer‐generated child body image array based on 3D scans of children (Jones et al., 2018; Ridley et al., 2024) to support better acknowledgement and understanding of child weight and a companion website. Control participants received the standard (non‐enhanced) NCMP letter, and nothing else. As such, the MapMe2 intervention closely resembled the MapMe1 intervention but (a) resources were refined, updated and further developed using parent involvement, and (b) all participants received a paper copy of the body scales *and* a QR link to the website – in MapMe1 parents received one or the other. The smaller, embedded study reported in this paper utilized an opt‐in subsample of the cRCT sample to better explore MapMe2 intervention mechanisms and outcomes in detail.

In the main MapMe2 cRCT, child BMI was measured at baseline by the NCMP in children aged 4–5 and 10–11 years, and again at 12‐month follow‐up to ascertain whether intervention allocation differentially influenced weight trajectory in BMI *z*‐score. Its findings are reported elsewhere (Jones, A.R., Shahrokhabadi, M.S., Basterfield, L., de Aguiar Greca, J.P., Sermin‐Reed, L., Patterson., M., Evans, E.H., Arauyo‐Soares, V., McSweeney, L., Robinson, T., Hiu, S., Tovee, M.J., Ells, L.J., Gahagan, A., Teare, M.D., Matthews, J.N.S. & Adamson, A.J., unpublished data. The opt‐in sub‐study included additional child‐ and parent‐reported behavioural and psychological measures alongside these weight measurements. We tested a logic model in which the intervention(s) led to improved parental acknowledgement and understanding of their child's weight, prompting increased self‐efficacy, action and coping planning, and precipitating changes in child energy balance behaviours, leading to weight change (S3 in Appendix S1). We therefore assessed parental child weight acknowledgement and understanding, objectively measured children's dietary intake and physical activity, and measured parent‐reported intentions, self‐efficacy, action and coping planning for supporting child energy balance behaviours. We also assessed a ‘dark’ logic model (S4 in Appendix S1) to check for potential negative effects for children, in whom the intervention's focus on weight might precipitate negative self‐evaluations, reduced health‐related quality of life, or attempts at weight loss through dietary restraint in older children. We consequently measured child self‐esteem, dietary restraint and quality of life. Overall, this study had the following primary aims:

- evaluate whether child behavioural and parental psychological determinants explained any observed intervention effects on child weight, testing a logic model; and
- evaluate whether children experienced any negative psychological sequelae from the intervention, testing a ‘dark’ logic model.

## METHOD

### Design

This sub‐study was nested within a larger cRCT of the intervention. The Newcastle University Research Ethics Committee granted ethical approval for this sub‐study (Ref: 2148/13605/2020).

**FIGURE 1:**
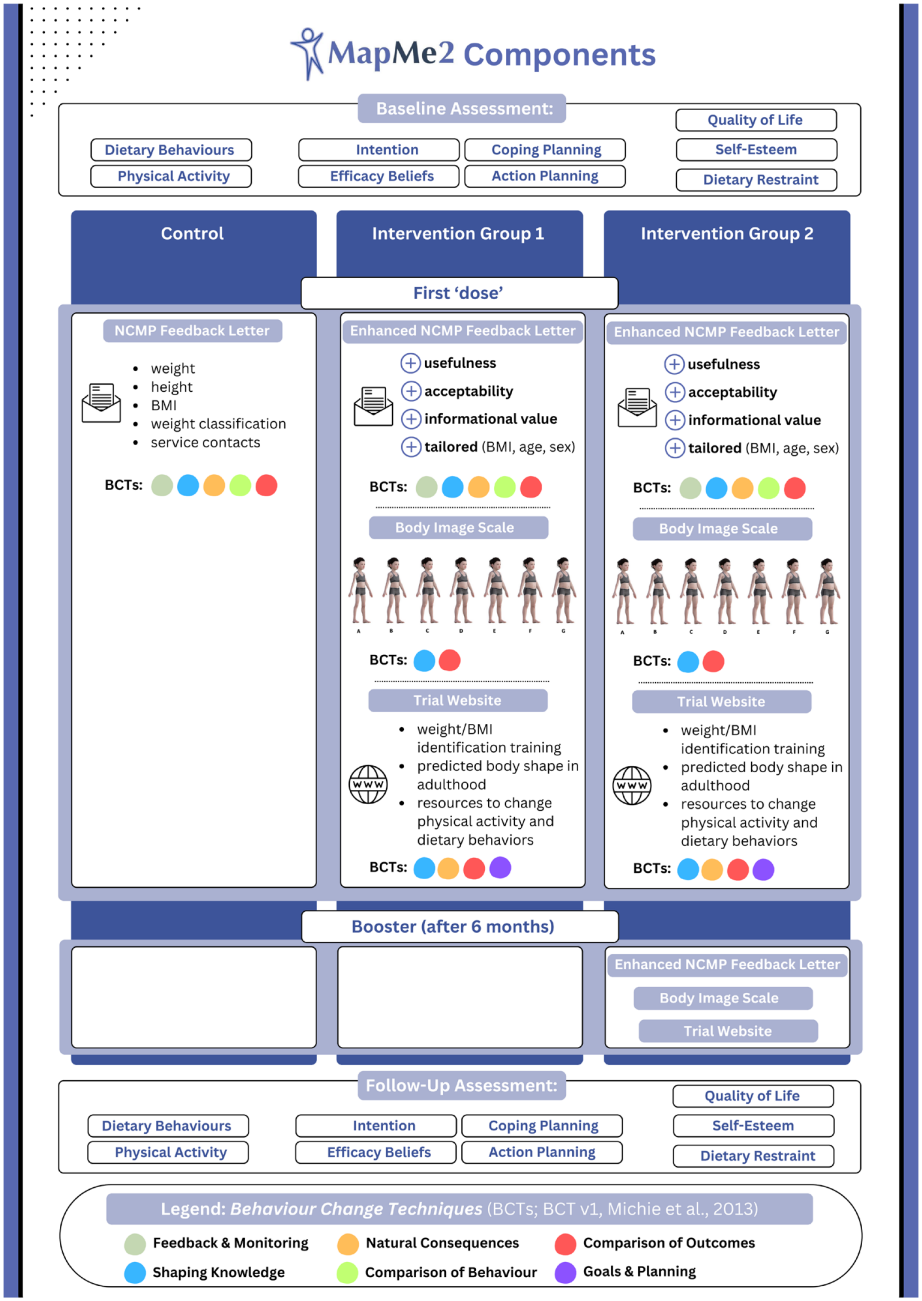
Schematic representation of MapMe2 intervention components and structure per group. Only *Intervention Group 2* received a booster after 6 months; Behaviour Change Techniques (BCTs) are indicated by colour (see legend); Body Image Scales depict the following weight centiles and classifications: 2nd (underweight); 25th, 50th and 75th (healthy weight); 91st (overweight); 98th and 99.6th (clinical obesity).

The main trial involved 10 Local Authorities in England and approximately 57,300 children at two time points 12 months apart, aged 4–5 or 10–11 at baseline (see ISRCTN12378125; https://doi.org/10.1186/ISRCTN12378125). Participants were cluster randomized in a ratio of 1:1:1 to one of three arms at the school level. Each local authority was assigned to two of three trial conditions. After the child had been weighed and measured (as per routine NCMP procedures), their household received the following materials by post depending on condition (see also Figure 1):

1. Control: Standard child weight feedback results letter informing them of their child's weight, height and weight classification, with details of any local child weight services.
2. Intervention 1: An enhanced feedback letter, the body image scale and supporting information, and a link to the trial website.
3. Intervention 2: Intervention 1, plus their results letter and another copy of the intervention materials 6 months after the first ‘dose’.

### Participants

Participants were 502 schoolchildren and their families who opted into the sub‐study from four of 10 English local authorities participating in the main trial and provided usable data from baseline and follow‐up. 51.03% were aged 4–5 (Reception; 45.08% boys) and 48.97% were aged 10–11 (Year 6; 46.52% boys) at baseline.

Participants were invited to the sub‐study via recruitment packs sent to home addresses with the NCMP pre‐measurement letter. This letter is sent to all eligible parents/carers to inform them that their child will be part of the NCMP unless they withdraw. The pack included an information leaflet, a consent form and a pre‐paid return envelope. Parents/carers interested in substudy participation provided active written consent for themselves and their child(ren). The sample in this study constitutes 3% of families invited. Two of the four local authorities offered weight management support services for children with higher weights, regardless of group allocation.

### Intervention

All parents/carers in the study received a letter providing detailed feedback on their child's weight status, as part of current NCMP procedures. Parents/carers receiving Intervention 1 received a paper copy of the body image scales specific to their child's age and sex and an ‘enhanced’ feedback letter with a QR code link to the study website once, whereas parents/carers receiving Intervention 2 received another copy of the scales and a ‘booster’ letter 6 months post‐measurement reminding them of the results and MapMe2 website.

Based on previous evidence synthesis and expert consensus identification, we selected the most promising and applicable BCTs for healthy weight services to support families with children 4–11 (Wehling et al., 2020; Figure 1). These targeted pre‐ as well as post‐intentional processes. First, based on social cognitive theories, procedural as well as outcome‐related knowledge and understanding, attitudes, and efficacy beliefs were assumed to increase intentions. Second, feedback, planning, and monitoring‐related BCTs were included to support the translation of such intentions into behaviour through action and coping planning as well as efficacy beliefs (TIDieR checklist (Hoffmann et al., 2014) and full BCT (Michie et al., 2013) description: S2a and S2b in Appendix S1). The content of these letters was additionally tailored to the weight classification of the child(ren) to better reflect parents' potential reaction to the information as well as to provide targeted advice and guidance. The content of the body image scales (both in paper form, and interactively via the website) was designed to enhance parental understanding of the range of child body weights and their corresponding visual features. This, in turn, aimed to prompt reflection on the correspondence between the figures and the child weight feedback given, and to provide feedback on the consequences of the energy balance behaviours discussed in the letter (Evans et al., 2025; Jones et al., 2023).

#### Body image scales

Both interventions included child body image scales showing children's bodies at seven different BMI points separately for each age group and gender (2nd, 25th, 50th, 75th, 91st, 98th and 99.6th centiles; Maalin et al., 2021). These used photorealistic CGI stimuli (Figure 1) based on 3D surface body scans of children, the development and validation of which is described elsewhere (Evans et al., 2025; Jones et al., 2018; Ridley et al., 2024). Stimuli were presented at a three‐quarter viewing angle because weight judgements for figures are more accurate in this orientation (e.g., Cornelissen et al., 2018; Evans et al., 2023). Accompanying text explained that the pictures showed body shapes of children of the same sex and age as the recipient's child and could be used to help understand child weight and recognize changes over time for one's own child(ren).

#### Study website

Both interventions included access to a study website (www.mapmeuk.co.uk, developed by *Antbits*) via a QR code in the enhanced feedback letter, with a unique household log‐in to ensure we could accurately track website usage by group allocation. The website showed the same images as the paper scales, but this time embedded in a 3D interactive tool that parents/carers could use to understand if their perception of their child's weight was correct or not, and which enabled them to visualize their child's simulated weight/BMI into the future. There, users could view and rotate the child's body images and enter their child's measurements to see the corresponding image. Parents/carers could visualize their child's body shape/size as a young adult if their BMI centile remained the same (they could also visualize changes to the BMI centile by changing weight/height input). Information was provided on the consequences of child's overweight and underweight, and external resources for healthy eating, physical activity and weight support. These resources focused on planning a healthier diet and optimal levels of physical activity for the family and child. The BCTs involved in these have not been fully coded as these are optional third‐party resources (e.g. ‘Change4Life’) which are regularly updated in line with the latest research and with seasonal public health campaigns.

#### Enhanced feedback letter

Both interventions included an enhanced feedback letter specific to their child's weight classification, which informed parents/carers of their child's weight, height, BMI and weight classification.

Adapted from the standard NCMP template letter and developed with iterative parent/caregiver input, it had additional features that aimed to increase its usefulness, acceptability and informational value for parents/carers, and enhance parental engagement:

- a reduced reading age and clearer language;
- a statement acknowledging that finding out one's child has a weight in the underweight/(very) overweight range can be difficult and unexpected, but help is available;
- an acknowledgement, when encouraging healthy dietary and physical activity behaviours, that families may already be taking these actions;
- encouragement to parents/carers to look at the included body image scales, which provided a guide to how children look at different weights, and an explanation that many people find it hard to tell a child's weight by looking;
- encouragement to parents/carers to visit the MapMe2 website (via a QR code) to check whether parents'/carers' perceptions of their child's size/shape matched their weight classification, and to access additional information, advice, and support with child growth and health;
- for parents/carers whose children had a weight below or above the recommended range, extra encouragement to contact the local NCMP team (‘we are looking forward to your call and talking to you about free local support you may want to use’).

The ‘booster’ letter, sent 6 months post‐measurement to parents/carers in Intervention group 2 only, again encouraged parents/carers to view the child body image scales to understand the range of child weight. It also invited parents/carers to visit the website again to (if wished) enter their child's up‐to‐date weight and height, engage with the intervention online resources, and access external resources supporting healthy weight.

In summary, the major distinction between the control and intervention letters was the inclusion of the printed Body Image Scale (age and sex appropriate) as well as a QR code linking to the MapMe2 website which provided additional external online resources. All participating families in every condition received letters providing child weight feedback, but the letters received by those in Interventions 1 and 2 were the enhanced versions as described above. The control group letters used the NCMP template and as such included a full link parents/carers could follow to reach appropriate local and national resources for healthy child weight.

### Outcome and process measures

#### Weight and height

Child weight and height were measured at baseline and 12 months according to NCMP procedure (*NCMP operational guidance*). Children were weighed and measured during the school day, privately, by trained school nursing teams and NCMP delivery staff, and were not informed of their measurements. Children wore light indoor clothing, without shoes and data were recorded to the nearest .1 kg (weight) and .1 cm (height). Measurements of children's height and weight alongside the child's sex and age were used to calculate their BMI *z*‐scores using the UK90 growth charts (Cole et al., 1995).

#### Energy balance behaviours

##### Dietary intake

Children's dietary intake was assessed using Intake24, an online dietary recall system, at baseline and 12 months, https://intake24.co.uk/ (V3, UK). Intake24 is an open‐source, multiple pass 24‐h dietary recall system (Foster et al., 2019). Participants completed 2×24 h recalls: parents/carers of younger children completed the measure, whereas older children self‐completed. While assessments were intended to occur on two weekend days, separated by a week, we had to also include some recalls across days of the week separated by ≤7 days.

##### Physical activity

Children's physical activity was assessed using 7‐day accelerometery at baseline and 12 months. Children wore triaxial ActiGraph wGT3x‐BT accelerometers (ActiGraph LLC, Pensacola, FL, USA) on their right hip continuously except showering, swimming or sleeping and parents completed concurrent wear timesheets. We recorded raw acceleration data at a 90 Hz sampling rate (Brønd & Arvidsson, 2016). Data initialisation and analysis used ActiLife (v.6.13.4), with segmentation into 15‐s epochs. Minimum wear time was 360 min/day, and non‐wear time was defined as 60 consecutive minutes of zero counts. Data from all participants with ≥2 valid days were analysed.

#### Parental weight acknowledgment and understanding

##### Weight acknowledgement

Parents/carers completed two questions assessing child weight classification perception, replicating the approach used by Jones et al. (2023). First, a single categorical question asked parents/carers ‘how would you describe your child's weight at the moment?’, with four possible responses: underweight, healthy weight, overweight, very overweight. These options matched the NCMP descriptors provided in feedback letters. Secondly, parents/carers used a dimensional visual analogue scale ranging from ‘extremely underweight’ to ‘extremely overweight’ to respond to the prompt ‘please mark the place on the line which best describes your child's weight’.

##### Weight understanding

To measure their understanding of child weight, parents/carers were asked to view the child body image scales appropriate to their child's age and gender and select the figure which represented the threshold for child overweight. This provided a dichotomous match‐mismatch measure of parental understanding.

#### Psychological and behavioural determinants: Questionnaire measures

Participating families completed a battery of questionnaires, listed in Table 1, on psychological and behavioural determinants of child weight at baseline and 12 months. Briefly, parents reported self‐efficacy, intentions, action plans and coping plans for supporting their child's health behaviours and seeking child weight support – the latter was optional and completed only by parents with concerns about their child's weight. All children self‐reported self‐esteem and older children reported dietary restraint. Older children reported quality of life and weight‐related quality of life and parents completed proxy measures for younger children.

**TABLE 1:** Parent and child questionnaire outcome measures completed at baseline and 12‐month follow‐up.

Construct	Measure(s)	Example item	Internal consistency
Parental self‐efficacy	Schwarzer and Renner (2000) (2 adapted items per behavioural domain)	I believe that I can support my child to be physically active during the next month (1 = strongly disagree, 5 = strongly agree)	n/a
Parental intentions	Sniehotta, Scholz, and Schwarzer (2005) (one adapted item per behavioural domain)	I intend to support my child to have a healthy balanced diet during the next month (1 = strongly disagree, 5 = strongly agree)	n/a
Parental action and coping planning	Sniehotta, Scholz, Schwarzer, Fuhrmann, et al. (2005) (three adapted items per behavioural domain)	*Action planning*: During the next month, I have a detailed plan on when my child will eat a healthy diet *Coping planning*: During the next month I have a detailed plan on what to do if something interferes with my plans to support my child to be physically active (1 = strongly disagree, 5 = strongly agree)	Baseline: *ω*_total:diet_ = .90; *ω*_total:PA_ = .96 Follow‐up: *ω*_total:diet_ = .93; *ω*_total:PA_ = .96 Baseline: *ω*_total:diet_ = .95; *ω*_total:PA_ = .96 Follow up: *ω*_total:diet_ = .96; *ω*_total:PA_ = .97
Child quality of life (generic; self‐reported by older, parent‐reported for younger children)	Child Health Utility 9D index (CHU‐9D; Stevens, 2012)	9 items describing states ranging from no impairment to significant impairment: e.g., ‘I don't feel upset today’ to ‘I feel very upset today’	Baseline: *ω*_total_ = .79 Follow‐up: *ω*_total_ = .71
Child weight‐related quality of life (self‐reported by older, parent‐reported for younger children)	Weight‐specific Adolescent Instrument for Economic Evaluation (WAltE; Oluboyede et al., 2017)	I struggle to concentrate on school work (1 = never, 5 = always)	Baseline: *ω*_total_ = .75 Follow‐up: *ω*_total_ = .67
Child self esteem (all self‐reported)	Lifespan Self‐Esteem Scale (LSES; Harris et al., 2018)	How do you feel about yourself? 5 pictorial response options from ‘really sad’ (downturned mouth, tears) to ‘really happy’ (open, upturned mouth)	Baseline: *ω*_total_ = .95; Follow‐up: *ω*_total_ = .94
Child dietary restraint (older children only)	Dutch Eating Behaviour Questionnaire‐Child: restraint subscale (DEBQC‐R; van Strien & Oosterveld, 2008)	Have you ever tried not eating after your evening meal to lose weight? (no, sometimes, yes)	Baseline: *ω*_total_ = .73; Follow‐up: *ω*_total_ = .75

### Procedure

Families had the choice to receive questionnaires either online using Qualtrics or by mail in paper form at baseline and 12 months. Paper questionnaires were returned using the provided stamped envelopes. Similarly, accelerometers and wear timesheets were delivered by mail and parents/carers returned them in a pre‐paid envelope, subsequently receiving a £5 voucher as thanks for the return. For the intake24 assessment, participants were sent links by email at both time points.

### Statistical analysis

Using the Lavaan package (Rosseel et al., 2012) in R (R Core Team, 2024) we estimated path analytical models to examine how group allocation affected (1) parents'/carers' acknowledgement of their child's weight classification, (2) children's BMI *z*‐scores through the hypothesised psychological and behavioural processes, and (3) any negative psychological sequelae.

**FIGURE 2:**
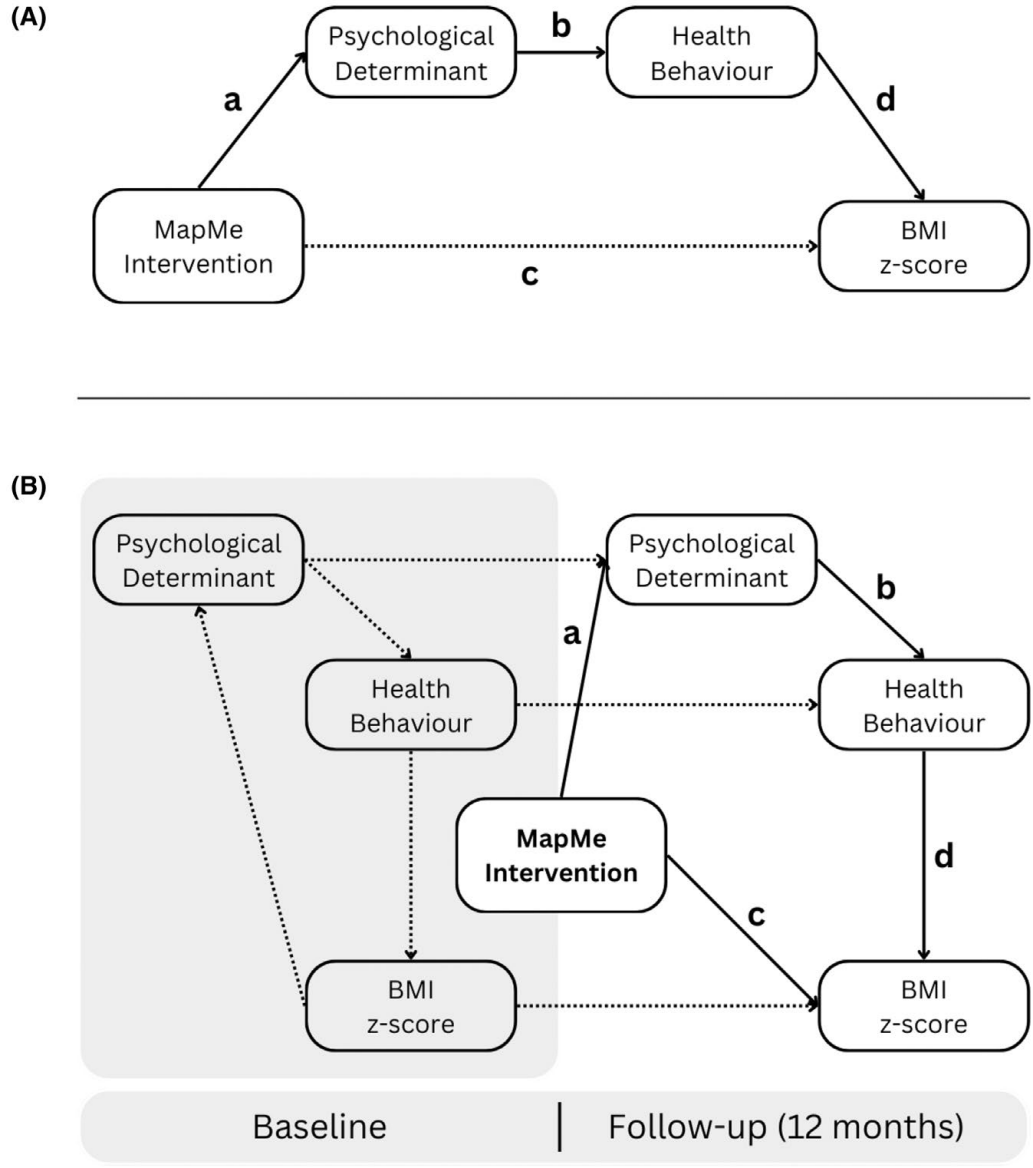
Conceptual model guiding the analyses. The upper panel (Panel A) outlines the proposed psychological and behavioural processes. The lower panel (Panel B) delineates how the variables of Panel A are proposed to operate over the two assessments (baseline, follow‐up at 12 months). The paths depicted in Panel B also represent the path diagrams that match the path analytic models for all health behaviours and psychological determinants. Path *a* captures the direct effect of the intervention on psychological determinants at 12 months. Path *b* captures the direct effect of the psychological determinants (assessed at 12 months) on health behaviours (assessed at 12 months). Path *d* captures the direct effect of the health behaviours on BMI *z*‐scores at 12 months. The composite Path *a* × *b* × *d* captures the indirect effect of the intervention on weight at 12 months (12 M) attributed to changes in psychological determinants and health behaviours (at 12 months; accounting for paths *c* and *d × e*). *Path c* captures the direct effect of the intervention on weight *z*‐scores at 12 months (12 M; accounting for other paths and baseline weight *z*‐scores). Beyond the hypothesised processes, Panel B also includes all auto‐regressive and cross‐lagged effects of the included variables at baseline and follow‐up (for example, the effect of weight *z*‐score at baseline on the score at follow‐up; all indicated by dashed paths).

For example, the path analytical models depicted in Figure 2B were specified to test all theory‐based psychological and behavioural mechanisms of the intervention (Figure 2A) while also accounting for auto‐regressive and cross‐lagged effects. Here, each model included one path for each of the interventions' effects (path a1 and a2). We followed the same modelling approach to examine parental perceptions and negative sequelae of the intervention.

Across all models, we adhered to the general intention‐to‐treat (ITT) principle by using full information maximum‐likelihood estimation to account for missing data (Arbuckle, 2013; Lee & Shi, 2021). We provide all analysis code, a synthetic dataset to facilitate reproducibility (OSF: https://osf.io/j7k3f/?view_only=27aca24dad8f4853b4a72d3f00b5b551) as well as a more detailed description of the analyses and in‐depth sensitivity analyses in a browseable web‐document here: https://cjoneshb.github.io/paper_mapme2/supplementary_materials.html

Fidelity was assessed by comparing the letters sent by local authorities to the template we provided. Intervention engagement per group was assessed by monitoring intervention website use using the unique login details provided in the enhanced feedback letter.

## RESULTS

### Sample characteristics at baseline, by group

The sub‐study sample was largely similar to that of the full trial, and no significant differences were detected between the two in terms of gender split, age and mean height/weight per age group. However, comparing the 10–11‐year‐olds, weight was differently distributed across categories: the full trial sample was on average heavier and more children with BMI *z*‐scores in the (very)overweight range were reported (most noticeably, 9.38%–11.11% of 10–11‐year‐olds in the sub‐study had very overweight BMI *z*‐scores, compared to 17.51%–18.34% in the full trial sample). Table 2 presents the sample characteristics for both samples, subdivided by age.

**TABLE 2:** Sample characteristics for the sub‐study sample and the full trial sample at baseline.

Sample	Sub‐study						Full trial					
	4–5 year olds			10–11 year olds			4–5 year olds			10–11 year olds		
Group	Control	Interv. 1	Interv. 2	Control	Interv. 1	Interv. 2	Control	Interv. 1	Interv. 2	Control	Interv. 1	Interv. 2
*N*	73	43	77	67	46	74	8340	9412	9657	8909	10,460	10,522
Girl	49.32%	55.81%	59.74%	56.72%	47.83%	54.05%	48.25%	50.36%	49.60%	49.44%	49.53%	48.49%
Boy	50.68%	44.19%	40.26%	43.28%	52.17%	45.95%	51.75%	49.64%	50.40%	50.56%	50.47%	51.51%
Age Mean (SD)	5.01 (.72)	5.01 (.30)	5.04 (.81)	10.79 (1.12)	10.98 (.34)	10.91 (.73)	4.99 (.75)	5.03 (.76)	5.05 (.60)	10.82 (1.53)	10.90 (1.52)	10.96 (1.27)
Height (cm) Mean (SD)	110.08 (6.66)	111.55 (6.17)	110.59 (6.96)	145.91 (9.61)	148.44 (7.13)	146.05 (6.95)	110.51 (5.26)	110.66 (5.20)	110.61 (5.28)	147.35 (7.55)	147.80 (7.43)	147.68 (7.62)
Weight (kg) Mean (SD)	19.53 (4.05)	19.82 (2.94)	19.39 (3.40)	39.11 (8.75)	41.20 (9.41)	38.26 (7.76)	20.02 (3.43)	20.06 (3.33)	19.97 (3.44)	43.53 (11.69)	43.59 (11.63)	43.50 (11.81)
BMI *z*‐score Mean (SD)	.21 (.92)	.19 (.77)	.08 (.87)	.28 (.98)	.37 (1.10)	.07 (1.14)	.41 (1.10)	.42 (1.05)	.37 (1.10)	.73 (1.31)	.69 (1.29)	.68 (1.31)
Weight category												
Underweight	0%	1%	1%	0%	0%	0%	1.10%	.69%	.97%	1.34%	1.29%	1.55%
Healthy weight	80.28%	87.18%	81.08%	79.69%	75.56%	79.71%	77.95%	79.76%	80.13%	61.44%	63.77%	63.40%
Overweight	12.68%	10.26%	13.51%	10.94%	13.33%	10.15%	8.97%	9.40%	8.82%	14.19%	13.85%	13.82%
Very overweight	7.04%	2.56%	4.05%	9.38%	11.11%	10.15%	7.18%	6.66%	6.69%	18.34%	17.51%	17.77%

### Engagement with website

We collected anonymised trial website usage data as an indicator of engagement with this intervention component. Six participants allocated to Intervention 1 accessed the website, as did 14 participants in Intervention 2, indicating a very low level of engagement. While we explore potential reasons for this else where McSweeney, L., Jones, A.R, Basterfield. L, Greca, J.P.A., Patterson, M., Adamson, A.J., unpublished data), these very low engagement metrics highlight that all potential intervention effects would most likely be due to the exposure to the enhanced letters and/or printed body image scales. This also affects which BCTs parents/carers saw, as goals and planning‐related BCTs specifically were included on the website.

### Changes in parental acknowledgement and understanding of child weight

**FIGURE 3:**
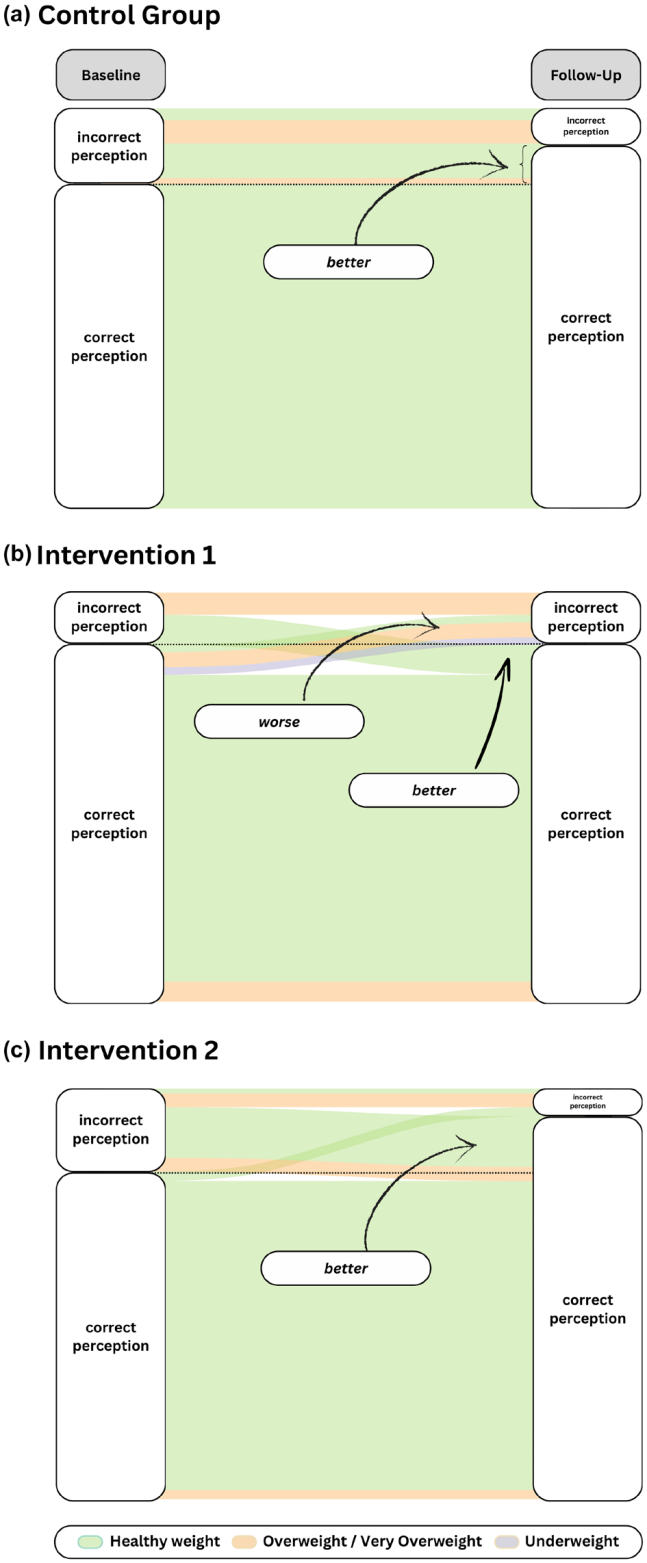
Alluvial plots depicting changes in parental perceptions of child weight by group (a) control, (b) intervention 1, (c) intervention 2 from baseline (left) to follow‐up assessment (right). Relative frequencies are coloured by weight classification (see legend below) and are calculated relative to the full size of the respective group.

At baseline, 81.16% of the parents/carers in the control group, 85.71% in the Intervention 1 group, and 80.42% in the Intervention 2 group assigned the correct weight classification to their child (Figure 3). At follow‐up, the parents/carers in the control group (89.86%; McNemar's *χ* ^2^ = 4.1667, *df* = 1, *p* = .041) as well as those in the Intervention 2 group showed significantly more accurate classification (88.46%; McNemar's *χ* ^2^ = 7.56, *df* = 1, *p* = .006). Those in the Intervention 1 group showed no significant changes (84.72%; McNemar's *χ* ^2^ = 0, *df* = 1, *p* = 1) in assigning their child's weight status.

Although parents'/carers' acknowledgement improved on average, there was no *significant effect of any group allocation* on the ratio of correct to incorrect acknowledgement of child weight classification at follow‐up, controlling for baseline (Intervention 1: *β* = −.11, SE = .06, *p* = .060; Intervention 2: *β* = −.03, SE = .06, *p* = .670; the model did not demonstrate a good fit to the data: *χ* ^2^(3) = 109.706, *p* = <.001, CFI = .275, RMSEA = .278).

**FIGURE 4:**
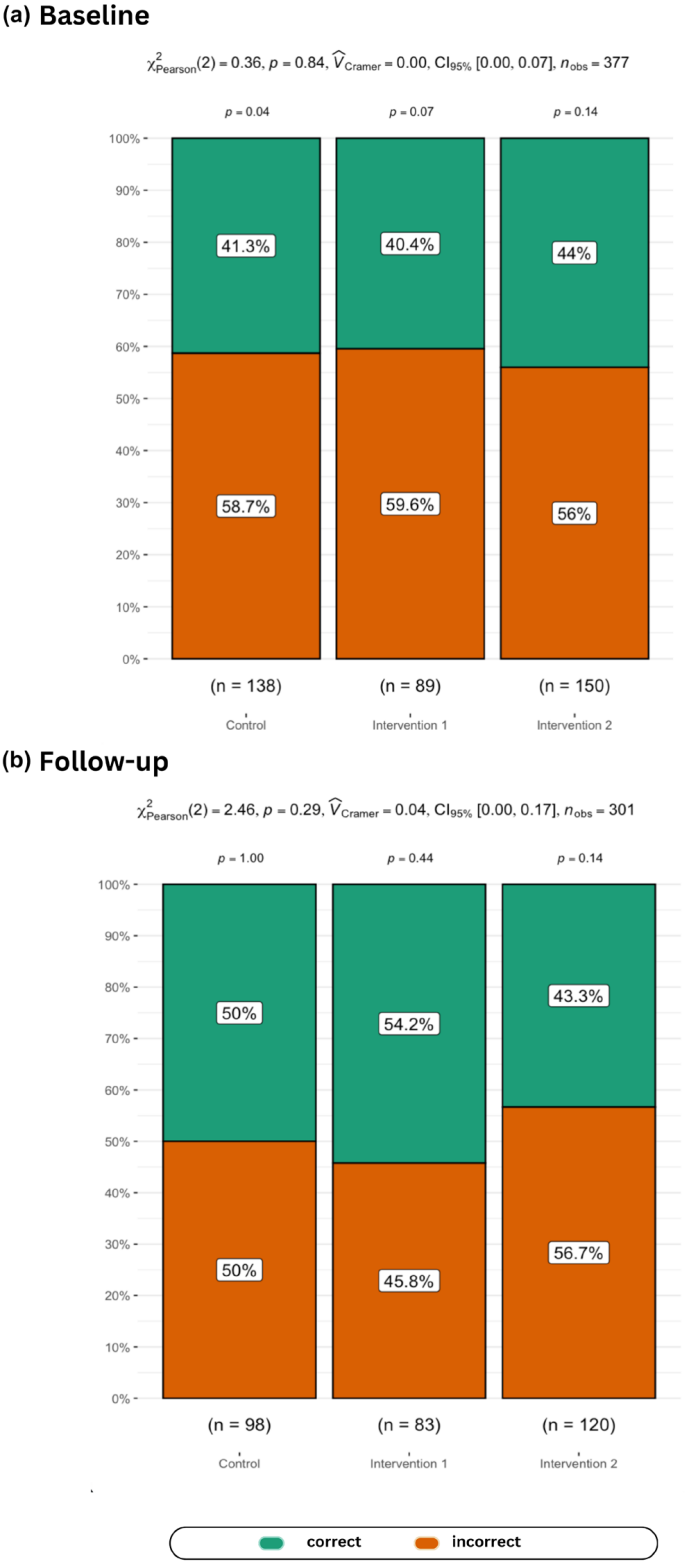
Relative frequencies for parental classification accuracy for child overweight/very overweight on a pictorial visual scale (provided in Figure 1) at baseline and follow‐up.

Regarding parental understanding of child weight, as Figure 4 shows, at baseline 40.4%–44% of the parents/carers correctly identified child images showing a BMI in the (very) overweight range using the MapMe figures (see Figure 1), while 43.3%–54.2% did so at follow‐up. Being allocated to one of the intervention groups did not improve discernment (Intervention 1: *β* = .04, SE = .06, *p* = .549; Intervention 2: *β* = −.07, SE = .07, *p* = .272).

### Psychological processes

In line with the findings of the main cRCT paper (Jones, A.R., Shahrokhabadi, M.S., Basterfield, L., de Aguiar Greca, J.P., Sermin‐Reed, L., Patterson, M., Evans, E.H., Araújo‐Soares, V., McSweeney, L, Robinson, T., Hiu, S., Tovée, M.J., Ells, L.J., Gahagan, A., Teare, M.D., Matthews, J.N.S. & Adamson, A.J. unpublished data), we found no effect of either Intervention 1 or Intervention 2 on BMI *z*‐scores at follow‐up when controlling for baseline scores. Overall, BMI *z*‐scores were stable across measurement points (*β* = .92, SE = .01, *p* < .001; see also S1 in Appendix S1). As not only BMI *z*‐scores but also most psychological and behavioural determinants remained largely stable, we find no coherent effects of the interventions on the proposed processes (Table 3).

**TABLE 3:** Integrated summary of path analytic models specified as depicted in Figure 2 estimated separately for each health behaviour and determinant.

Behaviour	Healthy eating		Unhealthy eating		F & V intake		MVPA		Sedentary behaviours	
	Est.	SE	Est.	SE	Est.	SE	Est.	SE	Est.	SE
Regressions										
I1 → IN (a1,1)	−.07	.07	−.07	.07	−.10	.07	−.06	.06	−.03	.05
I1 → AP (a2,1)	−.04*	.06	−.04	.05	−.05	.06	−.14***	.06	−.14*	.06
I1 → CP (a3,1)	.03	.06	.03	.06	.02	.06	−.00	.06	−.00	.06
I1 → EB (a4,1)	−.08	.06	−.08	.06	−.09	.06	.00	.06	.01	.06
I2 → IN (a1,2)	−.01	.06	−.01	.06	−.04	.06	−.06	.06	−.06	.05
I2 → AP (a2,2)	−.16*	.06	−.16**	.06	−.17**	.06	−.17***	.06	−.17**	.06
I2 → CP (a3,2)	−.11	.06	−.11	.05	−.12*	.06	−.11	.06	−.11*	.06
I2 → EB (a4,2)	−.09	.05	−.08	.06	−.10	.06	−.04	.06	−.03	.06
IN → HB (b1)	−.11	.06	−.06	.08	.34***	.08	.09	.05	−.02	.04
AP → HB (b2)	−.03	.06	.00	.08	.12	.07	.02	.06	−.01	.04
CP → HB (b3)	−.04	.07	−.05	.07	.09	.07	−.03	.06	.00	.04
EB → HB (b4)	−.00	.06	.01	.08	.21**	.07	.12	.06	−.03	.04
HB → Weight (d)	.02	.02	.00	.02	−.03	.03	−.06*	.03	.06*	.03
I1 → Weight (c)	.02	.02	.02	.02	.02	.02	.02	.02	.02	.02
I2 → Weight (c)	.02	.02	.02	.02	.02	.02	.02	.02	.02	.02
BW → Weight	.92***	.01	.92***	.01	.92***	.01	.92***	.01	.92***	.01
Model fit										
CFI	.865		.872		.876		.884		.904	–
RMSEA	.123		.120		.119		.121		.117	–
AIC	9253		8528		11,602		7083		8166	–
BIC	9371		8645		11,719		7201		8283	–
Model significance										
*N*	483		483		483		486		486	–
*χ* ^2^	133.30		126.53		125.74		130.50		122.97	–
df	16		16		16		16		16	–
*p*	<.001		<.001		<.001		<.001		<.001	–

*Note*: Not all paths of each model are included in this table. Please find all estimated paths in the Appendix S1. For all estimated models, missing values were handled by estimating the most likely population parameters based on the sample data (full information maximum likelihood). I1 = Intervention 1, I2 = Intervention 2, IN = Behavioural Intentions, AP = Action Planning, CP = Coping Planning, EB = Efficacy Beliefs, HB = (respective) Health Behaviour, BW = Baseline Weight; for example, paths *a1* and *b1* are sub‐paths of indirect path of each intervention on weight *z*‐scores through behavioural intentions (a1) and the respective health behaviour (b1).

*p < .05, **p < .01, ***p < .001.

*On a psychological level*, surprisingly, both interventions significantly decreased parents' action planning for activity‐related behaviours. In addition, Intervention 2 decreased parents' action planning for all diet‐related behaviours. Allocation to Intervention 2 also led to decreased parental coping planning for fruit and vegetable intake and sedentary behaviours. No other psychological determinants were affected by participants' group allocation. *On a behavioural level*, we found that stronger parental intentions (path a: *β* = .34, SE = .08, *p* < .001) as well as stronger efficacy beliefs (path a: *β* = .21, SE = .07, *p* < .01) were associated with higher child fruit and vegetable intake. Regarding children's weight, MVPA was negatively (path d: *β* = −.06, SE = .03, *p* < .05), while sedentary behaviours were positively, associated with BMI *z*‐scores (path d: *β* = .06, SE = .03, *p* < .05). No other health behaviours were associated with changes in BMI *z*‐scores.

Summarizing these findings, we see heterogenous and non‐coherent effects as the two interventions only exerted an influence on action planning and coping planning, while only efficacy beliefs were associated with key health behaviours – but action and coping planning were not. Still, even such coherent composite paths (path *a* × *b*) for diet‐related behaviours would not have brought about the intended changes in children's weight as only activity‐related behaviours showed a significant effect on BMI *z*‐scores. This was also evident across all models as no composite path was significant. Overall, the models demonstrated a poor fit to the data with CFI values ranging from .865 to .904, and RMSEA values from .117 to .123.

### Negative consequences

Similar to the assumed but non‐significant effects through psychological and behavioural determinants reported above, fortunately in this case, we also find no effects of the intervention on negative sequelae (i.e., reduced self‐esteem or increased dietary restraint; Table 4). While we find no intervention effect (path a), both indicators of quality of life remained stable over time, but neither self‐esteem nor dietary restraint were predicted by their baseline value (path b). Importantly, most models did not demonstrate a good fit to the data with CFI values ranging from .601 to .924 and RMSEA values from .057 to .099. The conclusions drawn from these models should thus be treated with caution (Table 4).

**TABLE 4:** Integrated summary of path analytic models predicting negative consequences (dark logic model, S4) as latent constructs.

	Model 1: Quality of life (CHU‐9D)		Model 2: Quality of life (WAltE)		Model 3: Self‐esteem (LSES)		Model 4: Dietary restraint (DEBQ‐C‐R)	
	Est.	SE	Est.	SE	Est.	SE	Est.	SE
Regressions								
I1 → UC	.08	.07	−.02	.07	.08	.08	−.13	.14
I2 → UC	−.00	.07	−.01	.07	−.01	.10	−.10	.14
BUC → UC	.69***	.11	.83***	.10	.10	.15	.53	.45
Model fit								
CFI	.703		.601		.924		.676	
RMSEA	.071		.099		.084		.057	
AIC	12,170		9854		5283		4437	
BIC	12,423		10,057		5411		4641	
Model significance								
*N*	470		470		464		469	
*χ* ^2^	569.59		574.28		144.17		259.56	
df	169		103		34		103	
*p*	<.001		<.001		<.001		<.001	

*Note*: Not all paths of each model are included in this table. Please find all estimated paths in the Appendix S1.

Abbreviations: BUC, Baseline Unintended Consequence; I1, Intervention 1; I2, Intervention 2; UC, Unintended Consequence.

*p < .05, **p < .01, ***p < .001.

Overall, therefore, the intervention did not differentially negatively affect children. Taking the sample as a whole (collapsed across allocated groups) we also found that receiving NCMP feedback did not appear to significantly worsen children's self‐esteem, dietary restraint, or quality of life, i.e., there was no overall group change from pre‐ to post‐measurement in these variables.

**FIGURE 5:**
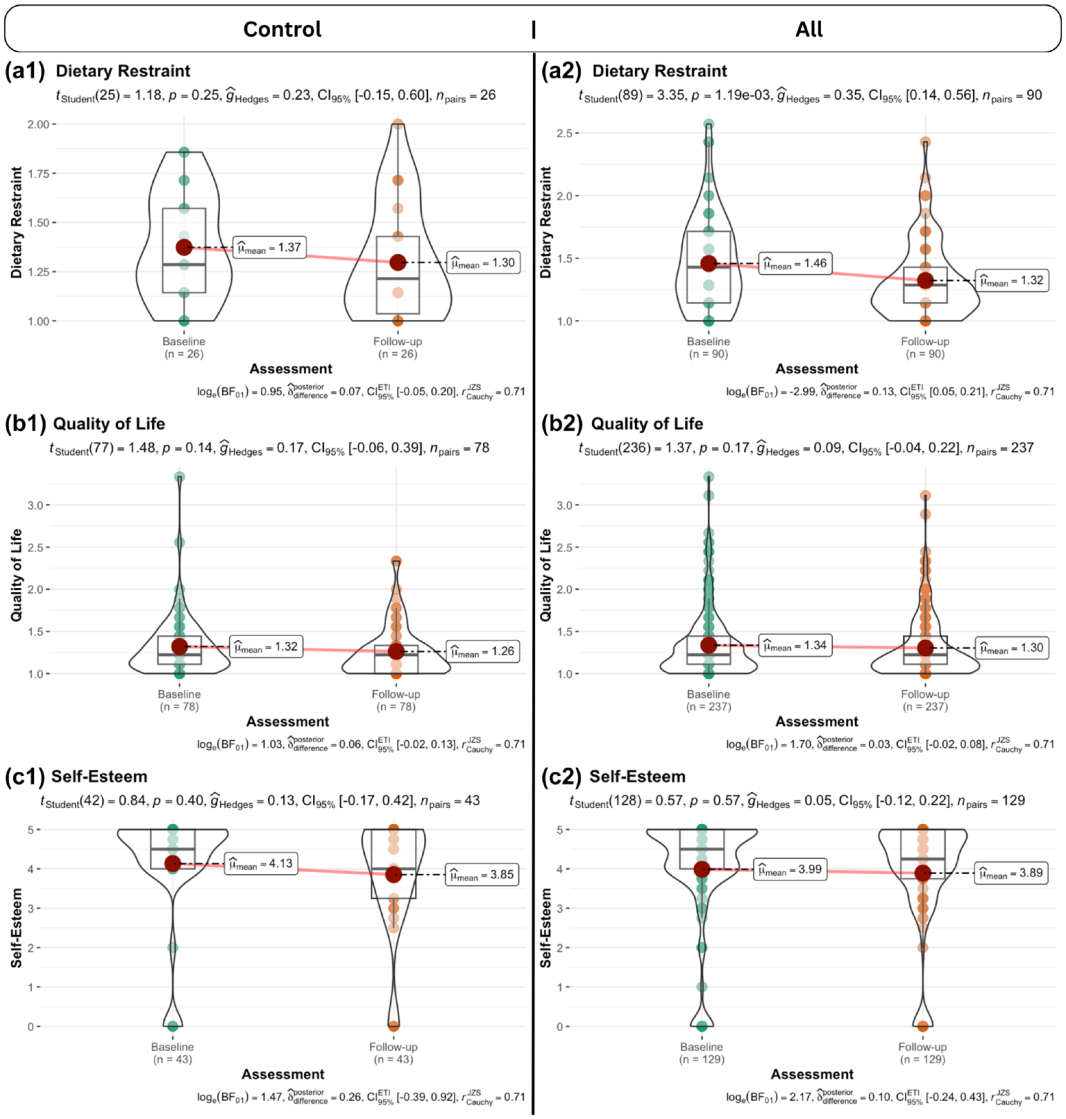
Comparison of baseline and 12‐month follow‐up assessments of negative sequelae (a) dietary restraint, (b) quality of life, and (c) self‐esteem) for control group (left) and all groups together (right). Depicted are different combined visualizations of the respective distributions (that is, violin‐, box‐, and jittered dot plots and means) Each plot includes inferential evidence on whether the respective null hypothesis should be rejected (above) or whether there is evidence in favour of the alternative hypothesis (below). Bayes Factors given below are on a natural logarithmic scale.

To examine whether the repeated weight assessment itself might increase negative sequelae, we also compared the respective means at baseline and follow‐up for the control group alone and all groups together (Figure 5 below). We found that dietary restraint decreased significantly when analysing all groups together, but not for the control group only (Figure 5a1,a2). While we also found decreases in quality of life and self‐esteem, these are not significant. Given the sample size and the potential keen interest in any evidence for the alternative hypotheses, we also provide Bayes Factors (BFs; on a natural logarithmic scale). Here, BFs mostly provide anecdotal to moderate evidence for the alternative hypotheses (Andraszewicz et al., 2015) that should be followed‐up with suitable designs and matching sample sizes.

### Intervention fidelity

We examined the extent to which the materials sent to parents/carers by the local authorities corresponded to the intervention template materials provided. Local authorities could not edit the body image scales, which were consequently delivered as planned. Local authorities edited the enhanced feedback letter to provide local phone numbers and addresses and add details of locally available resources and services for children and families; these additions were encouraged and expected. Analyses of the core body of the weight classification tailored letters sent to parents/carers (which should not have been edited) showed similarity with the templates that ranged from 97% to 100%, indicating very high fidelity.

## DISCUSSION

### Summary of findings

This sub‐study evaluated the mechanisms and psychological and behavioural outcomes of a very low‐intensity intervention to improve parental understanding and acknowledgment of child weight, nested within a larger cRCT. Neither intervention group showed differentially improved understanding/acknowledgement of child weight, in line with the findings of the main cRCT (MapMe2; Jones, A.R., Shahrokhabadi, M.S., Basterfield, L., de Aguiar Greca, J.P., Sermin‐Reed, L., Patterson, M., Evans, E.H., Araújo‐Soares, V., McSweeney, L, Robinson, T., Hiu, S., Tovée, M.J., Ells, L.J., Gahagan, A., Teare, M.D., Matthews, J.N.S. & Adamson, A.J., unpublished data). In keeping with this null effect, we also found that, overall, our hypothesised intervention mechanisms (based on the pre‐specified logic model) were not supported by the data. Children in the intervention groups did not show increased physical activity or improved dietary intake, and their parents/carers did not report positive changes in intentions, self‐efficacy, action or coping planning in relation to these behaviours. We also found that the intervention did not negatively affect children's wellbeing, specifically their child self‐esteem, dietary restraint, and quality of life. Overall results might be linked to very low levels of intervention engagement.

### Context of findings

This study is a valuable addition to the scant and heterogeneous existing literature about the effectiveness of low‐intensity educational/psychological interventions for parental acknowledgement and understanding of weight, and its relationship to weight outcomes. In keeping with several previous studies (Brown et al., 2020; Rune et al., 2015; Shen et al., 2020), providing additional information about the consequences of child weight and information about behaviour change did not result in differentially improved acknowledgement of child weight classification, over and beyond the effects of providing weight feedback to the whole sample. On this count, the findings accord with those of our previous trial (MapMe1; Jones et al., 2023) which did not find that the provision of the body image scales and guidance on how to use them affected parental acknowledgement in the intervention groups. In contrast to our current findings in MapMe2, our previous work in MapMe1 *did* find an effect on weight outcomes for children with overweight/obesity such that intervention recipients showed an attenuated trajectory of weight gain relative to the control group, using a broadly similar intervention albeit in a geographically concentrated smaller sample. To our knowledge, this previous trial is the only existing study in which the intervention influenced weight outcomes.

Regarding the intervention mechanisms in MapMe2, the data did not support the proposed composite paths from psychological to behavioural to weight variables. We saw isolated significant relationships, including, counter‐intuitively, decreased parental/caregiver action and coping planning for the intervention groups, which were not associated with any observed dietary/physical activity changes in their children. Higher physical activity was associated with lower child BMI *z*‐scores and sedentary behaviour was associated with higher BMI *z*‐scores. Higher parental intentions and self‐efficacy for healthy eating were associated with higher fruit and vegetable intake. However, overall, there was no coherent support for the hypothesised processes driving the intervention effects. A recent more complex and intensive intervention delivered directly to older adolescents in vocational schools targeted similar pre‐ and post‐intentional mechanisms of action to improve levels of physical activity and showed a similar null effect on most theorized determinants (Palsola et al., 2024). In contrast to the intended direct effects on adolescents' behavioural determinants, however, the proposed mechanisms of action in the present study targeted child weight indirectly through parental/caregiver behaviours.

We also found no detrimental effects of the MapMe2 intervention on child psychological wellbeing, as none of the tested composite paths were supported by the data, indicating that the intervention was not harmful to those who received it in the sub‐study sample. For researchers considering similar work in this area, this finding is reassuring given concerns raised about the effects of both the measurement and feedback processes on children (Jessen et al., 2023).

There are several potential explanations for the observed null findings for parental acknowledgement and understanding, and child weight outcomes, particularly in relation to the previous trial (MapMe1) which did see effects on child weight outcomes. Our intervention was particularly light‐touch compared to some previous interventions, involving no direct interactions with parents or children. A key difference between MapMe1 and MapMe2 was the use of active (MapMe1) versus opt‐out (MapMe2) consent procedures. Specifically, parents did not opt into the main trial so there was no initial active ‘buy in’ to the intervention or indeed, perhaps, awareness of it: sub‐study participants opted into the evaluation only. Intervention intensity was extremely low, and the postal intervention modality likely facilitated parental disengagement. This is particularly likely given the strong recent trend of parents/carers searching for health‐related information online (Kubb & Foran, 2020). Future interventions might thus leverage those digital modalities already most prominently used by parents/carers. However, in the case of MapMe2, these low levels of engagement also led to very few parents/carers engaging with the BCTs targeting post‐intentional processes that were only presented on the website. In addition, BMI *z*‐score was highly stable over the follow‐up period. Body weight is significantly biogenetically determined and also affected by so many variables that trajectory modification typically requires substantial family level input from weight support services (Bambra et al., 2015). This does not, however, explain why MapMe1 (Jones et al., 2023) found effects on weight outcomes using a very similar approach albeit with a small, more geographically concentrated and potentially more engaged sample (based on the assumption that parents that opt in to such studies may have higher levels of interest, awareness and motivation than those who do not). Another possible explanation for low engagement is timing: the current intervention was deployed immediately post COVID‐19 pandemic, which entailed multiple social, economic, educational and psychological burdens for UK families (e.g., Christie et al., 2022; Fosco et al., 2022) including increased child overweight/obesity (Santorelli et al., 2023).

Considering the sub‐study specifically, while the sample did not differ from the main MapMe2 trial sample regarding characteristics such as gender split, the full trial sample was on average heavier and included more children with BMI *z*‐scores in the (very)overweight range. This might not only have created differences between both samples but also indirectly reduced the power to detect small effects. Differences between letters for the control versus intervention groups in MapMe2 were necessarily subtle, given the need to align with a national template, although this was also the case in MapMe1 (Jones et al., 2023) in which intervention effects on weight *were* seen. In addition, our sub‐study findings demonstrate that parental engagement with the intervention website was extremely low. Low to moderate engagement might reasonably be expected given that most families were informed that their child had a healthy weight, thus reducing the perceived need to act or seek further information or support. However, other factors clearly supressed engagement further. Notably, the website did not provide instant access from the QR code: parents were asked to log in before use using a specific code. Whilst this enabled us to accurately track website use per intervention group and prevented individuals from outside the trial using the website, it presented an additional hurdle to use. Overall, therefore, low intensity and observed engagement alongside significant post‐pandemic disruption may partially explain our findings.

### Overall effects of the National Child Measurement Programme

We found that parental acknowledgement of their child's weight classification improved over time for the whole sample, suggesting that receiving feedback from the NCMP, in general, has a net positive effect on the parental acknowledgement of weight classification. To our knowledge, ours is the first paper to demonstrate this. Reassuringly, we also found that alongside this apparent improvement in parental acknowledgement, the self‐esteem and quality of life of children did not significantly deteriorate, and older children's levels of dietary restraint did not increase. This provides some reassurance to those with concerns about the impact of weight monitoring programmes themselves on children's wellbeing (e.g., Jessen et al., 2023). A caveat is that our sub‐study families opted in so potentially the most psychologically vulnerable children were not in our study. However, the sub‐study composition was similar to that of the main trial, making this explanation less likely. Another caveat is that all children in the MapMe2 trial were weighed and measured twice, 12 months apart, rather than once as per usual NCMP procedures ‐ as such their experiences were not identical. However, one would not expect repeated weighing to have a *lesser* effect on child wellbeing than a single occasion, so the finding of no detriment remains reassuring.

### Strengths and limitations

Except for the unbalanced number of children in the (very)overweight range, the characteristics of the sub‐study sample and the larger cRCT sample were similar, meaning that it is reasonable to draw broader conclusions about the intervention from our findings. We coherently examined a wide range of theoretically informed psychological and behavioural determinants (and processes) in children and their parents/carers, testing pre‐specified logic models for both desired and undesired mechanisms of impact.

While the study leveraged a longitudinal design, not all constructs were assessed in the temporal order that allows causal inferences. Specifically, we assessed all psychological/behavioural determinants of potential changes in BMI *z*‐scores contemporaneously at 12‐month follow‐up. Thus, we can only determine whether group allocation led to changes in these constructs, but not whether they were causally, and in line with the proposed model, linked to each other. This is a clear limitation of this study. In addition, the study originally aimed to recruit nearly 1200 children and families to participate, assuming an opt‐in recruitment rate of approximately 8% (as per Jones et al., 2023). In the event, the recruitment rate was substantially lower than this, at 3%. This low uptake may be due to the relatively time‐consuming nature of participating in this sub‐study, which was not remunerated and spanned a prolonged period. It may also be partially attributable to increased post‐pandemic familial disruption. This might also have contributed to the lower number of children in the (very)overweight range participating in the sub‐study which, in turn, reduces the generalizability of the findings reported here to the overall sample and population of interest.

## CONCLUSIONS

While this quantitative process and outcome evaluation neither showed the effects of the very low‐intensity intervention on parental understanding/acknowledgement of child weight nor on children's BMI *z*‐scores through the proposed mechanisms of action, it did not result in negative consequences either. Instead, our overall results suggest a net positive effect of the NCMP letters in general as parental acknowledgement of children's weight classification improved over time for the whole sample while self‐esteem and quality of life of children did not significantly deteriorate. Although the intervention could not facilitate further improvements, this provides some reassurance to concerns about the impact of weight monitoring programmes on children's wellbeing.

Development of this intervention necessitated making subtle refinements to an existing monitoring programme, forcing focus on reach, scalability, and pragmatic implementation (Holtrop et al., 2021). More research is needed regarding ways to facilitate parental/caregiver engagement with available behaviour change resources (e.g., within the NCMP letters). Future research might particularly focus on how to leverage sources of child weight information that parents are already using, such as social media, and might move away from postal delivery approaches. It is also important, in future research focused specifically on mechanisms, to ensure that measurement of psychological/behavioural variables and weight outcomes is done on a schedule that would permit sequential inferences between psychological determinants, behaviours, and weight outcomes.

## AUTHOR CONTRIBUTIONS

**Elizabeth H. Evans:** Conceptualization; methodology; writing – original draft; writing – review and editing; funding acquisition. **Tomos Robinson:** Methodology; investigation; data curation; writing – review and editing. **Mohadeseh Shojaei Shahrokhabadi:** Data curation; formal analysis; writing – review and editing. **Louisa Ells:** Conceptualization; funding acquisition; writing – review and editing. **Alison Gahagan:** Conceptualization; funding acquisition; writing – review and editing. **Dawn Teare:** Supervision; formal analysis; writing – review and editing. **Martin J. Tovée:** Conceptualization; funding acquisition; methodology; writing – review and editing. **Vera Araújo Soares:** Conceptualization; methodology; writing – original draft; writing – review and editing; funding acquisition. **Angela R. Jones:** Conceptualization; methodology; funding acquisition; project administration; supervision; writing – original draft; writing – review and editing. **Laura Basterfield:** Project administration; investigation; supervision; writing – review and editing. **Christopher M. Jones:** Writing – original draft; writing – review and editing; data curation; formal analysis. **Ashley Adamson:** Conceptualization; funding acquisition; writing – review and editing. **João Paulo de Aguiar Greca:** Data curation; methodology; project administration; supervision; writing – original draft; writing – review and editing. **Lorraine McSweeney:** Formal analysis; investigation; writing – review and editing. **Raenhha Dhami:** Methodology; formal analysis; investigation; writing – review and editing. **Letitia Sermin‐Reed:** Investigation; data curation; writing – review and editing. **Maddey Patterson:** Methodology; investigation; data curation; formal analysis; writing – review and editing.

## Supporting information


Appendix S1

## Data Availability

The data that support the findings of this study are available on request from the corresponding author. The data are not publicly available due to privacy and ethical restrictions. Instead, we provide all analysis code with a synthetic dataset that closely resembles the original data to facilitate reproducibility through the Open Science Framework (DOI: 10.17605/OSF.IO/J7K3F).
