# Supplementary material for: Mechanisms and outcomes of a very low intensity intervention to improve parental acknowledgement and understanding of childhood overweight/obesity, embedded in the National Child Measurement Programme: A sub‐study within a large cluster Randomized Controlled Trial (MapMe2)
Source: Br J Health Psychol. 2025 Feb 13;30(1):e12784. doi: 10.1111/bjhp.12784 (PMC11822560; doi:10.1111/bjhp.12784)

**Supplementary Materials**

S1. Comparison of BMI z-scores for both intervention groups at baseline and follow-up. *Note*. Depicted are violin-, box-, and jittered dot plots for BMI z-scores for web boost and web only intervention groups at A) *baseline*, and B) *follow-up*. Inferential statistics on between-group differences in means are given above each plot.


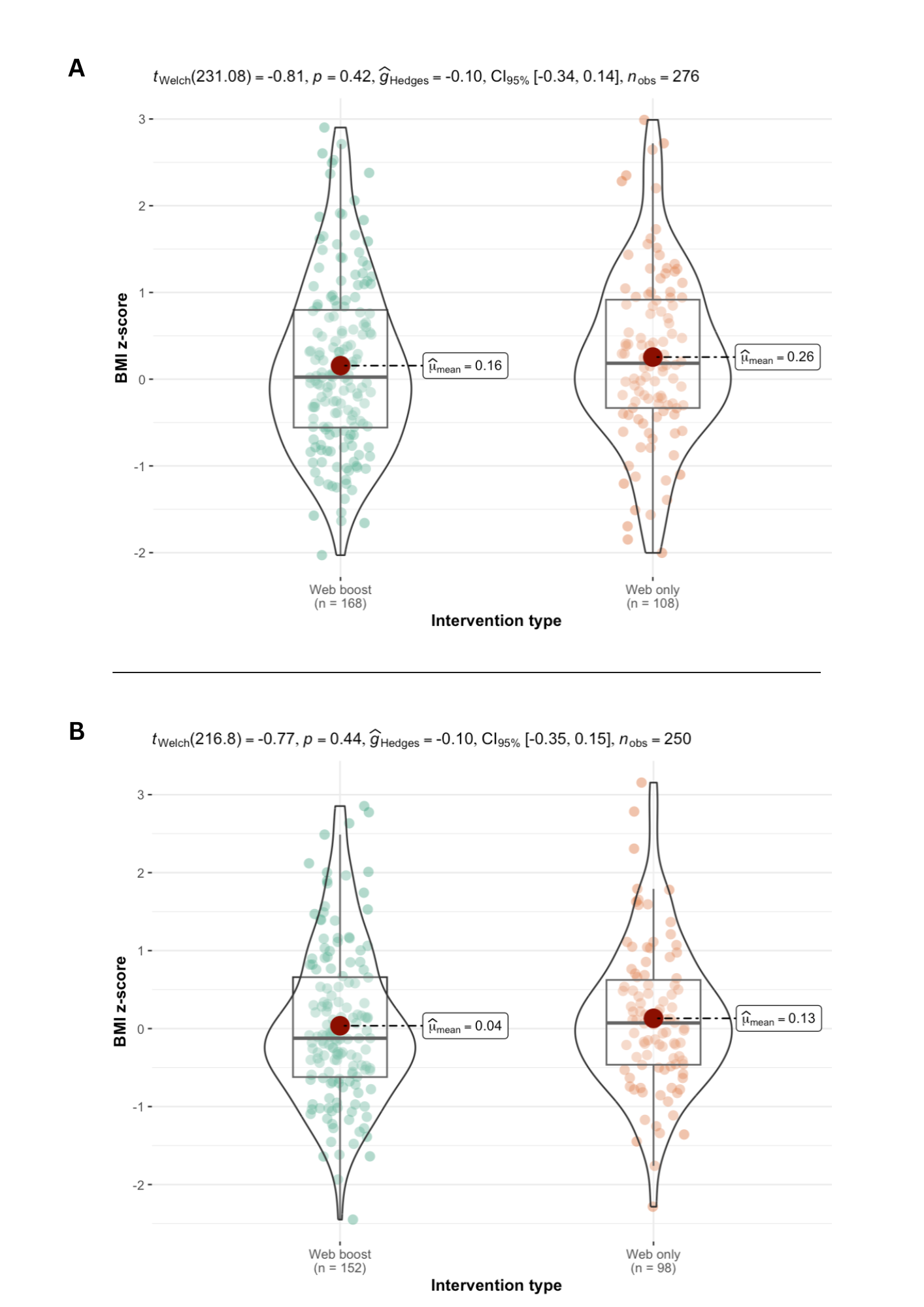


S2a

**
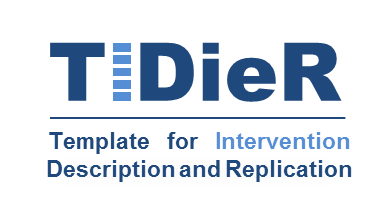
The TIDieR (Template for Intervention Description and Replication) Checklist*:**

Information to include when describing an intervention and the location of the information

| **Item number** | **Item** | **Where located **** | |
| --- | --- | --- | --- |
|  |  | Primary paper  (page or appendix  number) | Other ^†^ (details) |
|  | **BRIEF NAME** |  |  |
| **1.** | Provide the name or a phrase that describes the intervention. | ____8________ | ______________ |
|  | **WHY** |  |  |
| **2.** | Describe any rationale, theory, or goal of the elements essential to the intervention. | __5,6,8-10__ | ___S3_______ |
|  | **WHAT** |  |  |
| **3.** | Materials: Describe any physical or informational materials used in the intervention, including those provided to participants or used in intervention delivery or in training of intervention providers. Provide information on where the materials can be accessed (e.g. online appendix, URL). | __8-10_______ | ___S2b________ |
| **4.** | Procedures: Describe each of the procedures, activities, and/or processes used in the intervention, including any enabling or support activities. | __8-10_____ | _____________ |
|  | **WHO PROVIDED** |  |  |
| **5.** | For each category of intervention provider (e.g. psychologist, nursing assistant), describe their expertise, background and any specific training given. | ___n/a_______ | _____________ |
|  | **HOW** |  |  |
| **6.** | Describe the modes of delivery (e.g. face-to-face or by some other mechanism, such as internet or telephone) of the intervention and whether it was provided individually or in a group. | ____7,8______ | _____________ |
|  | **WHERE** |  |  |
| **7.** | Describe the type(s) of location(s) where the intervention occurred, including any necessary infrastructure or relevant features. | ____7,8______ | _____________ |
|  | **WHEN and HOW MUCH** |  |  |
| **8.** | Describe the number of times the intervention was delivered and over what period of time including the number of sessions, their schedule, and their duration, intensity or dose. | ___8,10______ | _____________ |
|  | **TAILORING** |  |  |
| **9.** | If the intervention was planned to be personalised, titrated or adapted, then describe what, why, when, and how. | ___8,9_______ | _____________ |
|  | **MODIFICATIONS** |  |  |
| **10.^ǂ^** | If the intervention was modified during the course of the study, describe the changes (what, why, when, and how). | ___n/a_______ | _____________ |
|  | **HOW WELL** |  |  |
| **11.** | Planned: If intervention adherence or fidelity was assessed, describe how and by whom, and if any strategies were used to maintain or improve fidelity, describe them. | ____14_______ | _____________ |
| **12.^ǂ^** | Actual: If intervention adherence or fidelity was assessed, describe the extent to which the intervention was delivered as planned. | _____17-18___ | _____________ |

** **Authors** - use N/A if an item is not applicable for the intervention being described. **Reviewers** – use ‘?’ if information about the element is not reported/not sufficiently reported.

† If the information is not provided in the primary paper, give details of where this information is available. This may include locations such as a published protocol or other published papers (provide citation details) or a website (provide the URL).

ǂ If completing the TIDieR checklist for a protocol, these items are not relevant to the protocol and cannot be described until the study is complete.

* We strongly recommend using this checklist in conjunction with the TIDieR guide (see *BMJ* 2014;348:g1687) which contains an explanation and elaboration for each item.

* The focus of TIDieR is on reporting details of the intervention elements (and where relevant, comparison elements) of a study. Other elements and methodological features of studies are covered by other reporting statements and checklists and have not been duplicated as part of the TIDieR checklist. When a **randomised trial** is being reported, the TIDieR checklist should be used in conjunction with the CONSORT statement (see [www.consort-statement.org](http://www.consort-statement.org)) as an extension of **Item 5 of the CONSORT 2010 Statement.** When a **clinical trial** **protocol** is being reported, the TIDieR checklist should be used in conjunction with the SPIRIT statement as an extension of **Item 11 of the SPIRIT 2013 Statement** (see [www.spirit-statement.org](http://www.spirit-statement.org)). For alternate study designs, TIDieR can be used in conjunction with the appropriate checklist for that study design (see [www.equator-network.org](http://www.equator-network.org)).

S2b. Behaviour change techniques (BCTs) *used in MapMe2 (Interventions 1 and 2), based on the BCTV1 taxonomy (Michie et al., 2013).*

The key **intervention** BCTs used were:

2. Feedback and monitoring

2.6. Biofeedback

4. Shaping Knowledge

4.1. Instructions on how to perform the behaviour (seeking support; physical activity)

5. Natural consequences

5.1. Information about health consequences

5.3. Information about social and environmental consequences (for overweight and very overweight)

5.6. Information about emotional consequences (for overweight and very overweight)

6. Comparison of behaviour

6.2. Social comparison

9. Comparison of outcomes

9.1. Credible sources (e.g., NCMP)

*The Body Image Scales were included in Interventions 1 and 2 in paper copy.*

*The MapMe2 website optionally provided the opportunity to visualise current child weight classification (shape/size) and visualise simulated adult weight for the child. Parents were required to follow a QR code to access the MapMe2 website if they wished: this afforded the inclusion of extra BCTs.*

4. Shaping knowledge

4.4. Behavioural experiment *(only possible if families would link the MapMe2 website)*

5. Natural consequences

5.2. Salience of consequences

9. Comparison of outcomes

9.3. Comparative imaging of future outcomes (Parents visualize the body size/shape of their future child as a young adult if their weight centile is maintained and can also visualise the effect of BMI (therefore centile) modification on their future size/shape).

Also, once reaching and proactively engaging the website QR code embedded in the experimental groups letter there was also the possibility of engaging with resources that would lead to the use of distinct BCTs such as:

1. Goals and Planning:

1.1. Goal setting (behaviour)

1.2. Problem solving

1.4. Action planning

*BCTs used in the NCMP booster letters were all those possible by the utilisation of the body image scale (BIS) as well as the same ones used during the experimental letter.*

*BCTs used in the NCMP* ***control letters*** *as well on the letters that were sent out to those children who have opted out of MapMe2, were:*

3. Feedback and monitoring

2.6. Biofeedback

4. Shaping Knowledge

4.1. Instructions on how to perform the behaviour (seeking support; physical activity)

5. Natural consequences

5.1. Information about health consequences

5.3. Information about social and environmental consequences (for overweight and very overweight)

5.6. Information about emotional consequences (for overweight and very overweight)

6. Comparison of behaviour

6.2. Social comparison

9. Comparison of outcomes

9.1. Credible sources (e.g., NCMP)

Also, once reaching and proactively engaging the website link embedded in the control group NCMP letter there was also the possibility of engaging with resources that would lead to the use of distinct BCTs such as:

1. Goals and Planning:

1.1. Goal setting (behaviour)

1.2. Problem solving

1.4. Action planning

The major distinction between the control and intervention letters was the inclusion of the printed Body Image Scale (age and sex appropriate) as well as a QR code linking to the MapMe2 website and additional external online resources. The enhanced intervention letters were designed with parental input to minimise recipient disengagement by (i) demonstrating awareness of potential parental/caregiver negative emotions associated with the child weight feedback, (ii) acknowledging that families may already be trying to make lifestyle changes and/or may already be optimising their child’s energy balance behaviours, (iii) encouraging participants to engage with freely available help and emphasising the local team’s interest in hearing from them.

The website involved a visual body image scale tool which parents/carers could use to understand if their perception of their child’s weight was correct or not, and which enabled them to visualise a simulation of their child’s weight/BMI into the future. It also provided resources in support of behaviour change for a healthier diet and higher levels of physical activity for the family and child. The BCTs involved in these have not been fully coded as these are third party resources (e.g. ‘Change4Life’). The control group letters did not have a QR code but a full link parents/carer could follow to reach a range of resources with similar content. Please note that the content on external websites and resources may have changed since this cRCT and substudy took place, so current content does not necessarily reflect what trial participants had the potential to see.

S3. Logic Model


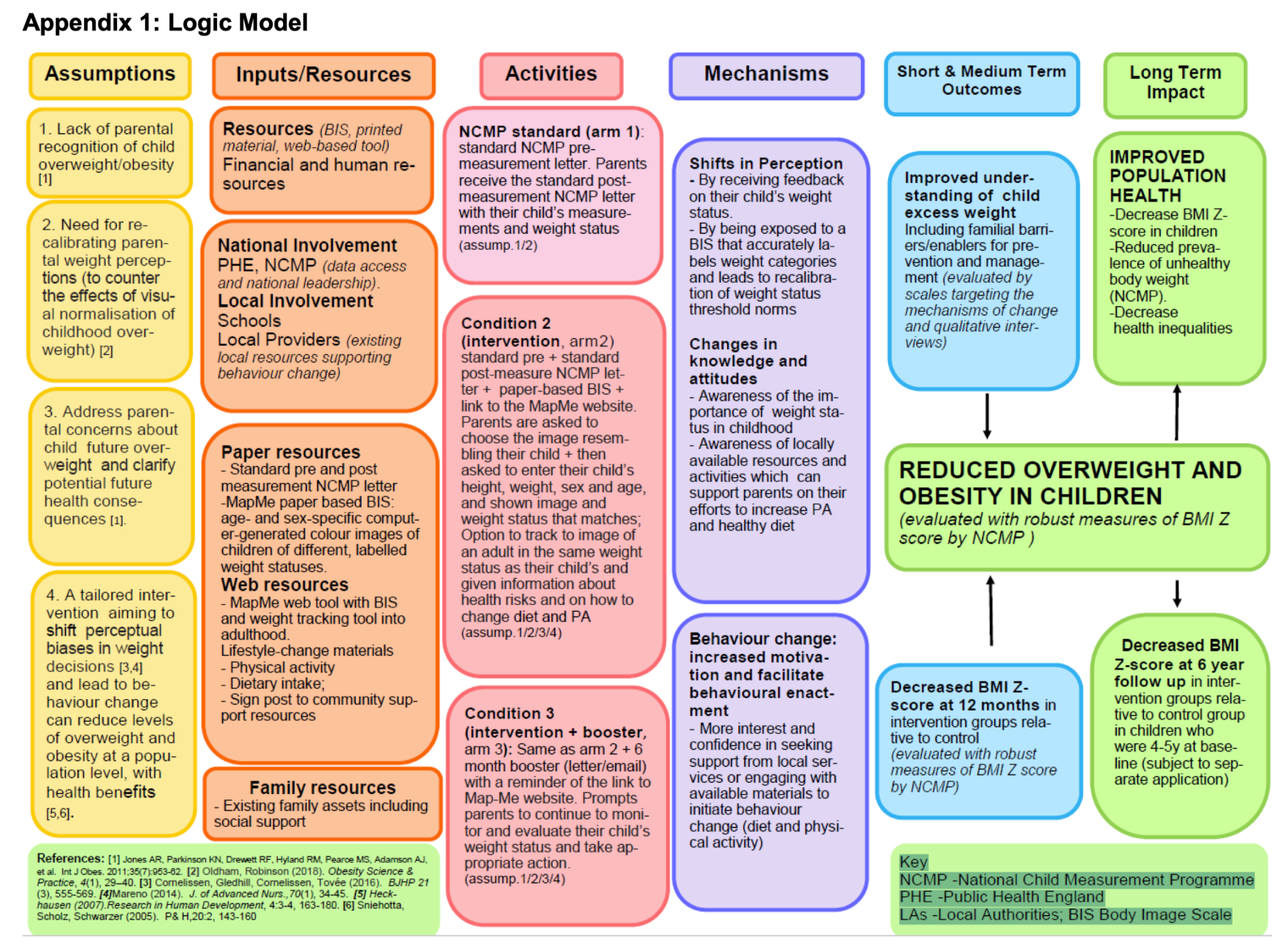


S4. Dark Logic Model


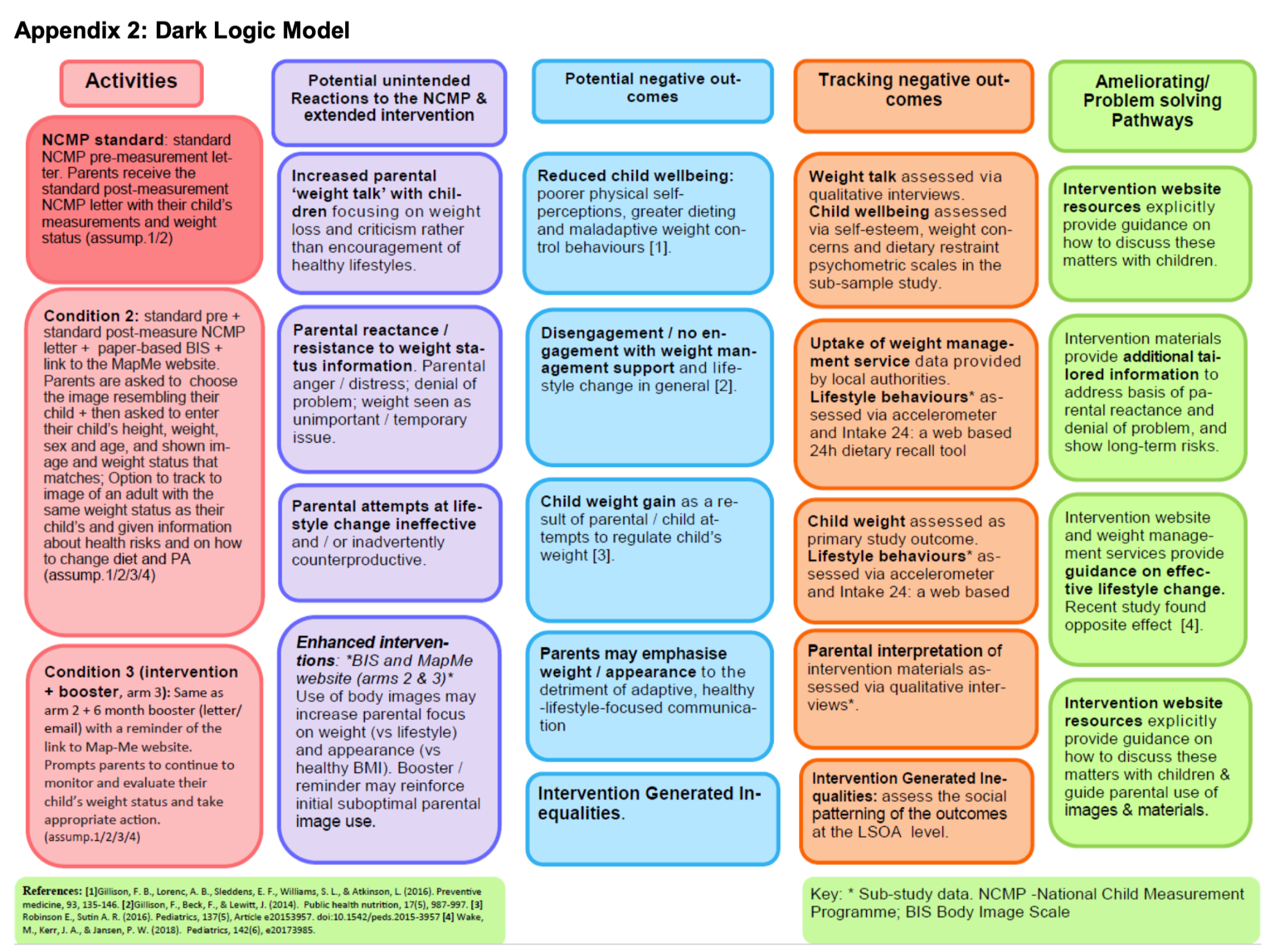

Supplement: Supplementary file 1 — Appendix S1 [file BJHP-30-0-s001.docx]
